# Lung CSC‐derived exosomal miR‐210‐3p contributes to a pro‐metastatic phenotype in lung cancer by targeting FGFRL1

**DOI:** 10.1111/jcmm.15274

**Published:** 2020-05-12

**Authors:** Li Wang, Jun He, Haoyue Hu, Li Tu, Zhen Sun, Yanyang Liu, Feng Luo

**Affiliations:** ^1^ Lung Cancer Center, Cancer Center, and State Key Laboratory of Biotherapy West China Hospital of Sichuan University Chengdu China; ^2^ Laboratory of Experimental Oncology State Key Laboratory of Biotherapy West China Hospital of Sichuan University Chengdu China

**Keywords:** exosome, lung cancer stem cell, metastasis, miR‐210‐3p

## Abstract

Lung cancer has the highest mortality rate among human cancers, and the majority of deaths can be attributed to metastatic spread. Lung cancer stem cells (CSCs) are a component of the tumour microenvironment that contributes to this process. Exosomes are small membrane vesicles secreted by all types of cells that mediate cell interactions, including cancer metastasis. Here, we show that lung CSC‐derived exosomes promote the migration and invasion of lung cancer cells, up‐regulate expression levels of N‐cadherin, vimentin, MMP‐9 and MMP‐1, and down‐regulate E‐cadherin expression. Moreover, we verified that these exosomes contribute to a pro‐metastatic phenotype in lung cancer cells via miR‐210‐3p transfer. The results of bioinformatics analysis and dual‐luciferase reporter assays further indicated that miR‐210‐3p may bind to fibroblast growth factor receptor‐like 1 (FGFRL1); silencing FGFRL1 enhanced the metastatic ability of lung cancer cells, whereas overexpressing FGFRL1 suppressed metastasis. Taken together, our results provide new insights into a potential molecular mechanism whereby lung CSC‐derived exosomal miR‐210‐3p targets FGFRL1 to promote lung cancer metastasis. FGFRL1 may be a promising therapeutic target in lung cancer.

## 1 INTRODUCTION

According to cancer statistics published in 2018, lung cancer is a leading cause of cancer‐associated morbidity and mortality worldwide.^1^ The most common cause of mortality in advanced lung cancer patients is metastasis, especially metastasis to the brain. Without treatment, the median overall survival of lung cancer patients with brain metastasis is only 1‐2 months.^2^ Therefore, exploring possible underlying molecular mechanisms involved with cancer metastasis, and developing new predictive targets and therapeutic strategies to suppress metastasis, seems to be of import for achieving clinical benefits in cancer patients.

Metastasis is closely associated with the epithelial‐mesenchymal transition (EMT),^3^ which endows cancer cells with enhanced migratory and invasive potential as well as phenotypic markers including E‐cadherin, N‐cadherin, vimentin, MMP‐9, MMP‐1 and MMP‐2.^4^, ^5^

MicroRNAs are non‐coding RNAs, 19‐22 nucleotides in length, which are closely associated with cancer progression, including metastasis. Song *et al* ^6^ found that miR‐26a‐5p promoted lung cancer metastasis. Jiang *et al* ^7^ demonstrated that miR‐19a‐3p potentiates hepatocellular carcinoma metastasis. Studies of breast cancer and cervical cancer have shown that microRNAs may foster metastasis by enhancing migratory and invasive abilities and altering the expression of EMT‐associated proteins such as E‐cadherin, N‐cadherin and vimentin.^8^, ^9^

Exosomes are extracellular vesicles (diameter 30‐150 nm) that are secreted by all living cells.^10^ They express specific surface markers, such as CD63, CD81 and Tsg101, and contain several types of bioactive materials, including microRNA, protein and DNA.^11^ Recent reports suggest that tumour microenvironment‐derived cells such as cancer‐associated fibroblasts (CAFs), myeloid‐derived suppressor cells (MDSCs) and mast cells may communicate with cancer cells and enhance their metastatic ability through the transfer of special microRNA encapsulated in exosomes.^12^, ^13^, ^14^ The metastatic potential of cancer cells may therefore be regulated by other types of cells in the tumour microenvironment through the transfer of exosomes carrying pro‐metastatic microRNAs.

Cancer stem cells (CSCs) are a small subset of heterogeneous cells found in tumour tissue or cultured cell lines. Previous studies have suggested that lung CSCs may be isolated and enriched from surgically resected tumour tissue or lung cancer cell lines such as A549, PC‐9 and H460.^15^, ^16^ These cells display obvious morphological differences from their parental lung cancer cells and express a stemness phenotype that includes enhanced ability for self‐renewal, resistance to chemo/radiotherapy, high expression of stemness‐associated markers such as CD133, ALDH1, Sox2 and Nanog, and increased metastatic potential. Due to these specific features, lung CSCs were considered to be the main mediators of lung cancer metastasis and recurrence.^17^ One recent study of renal carcinoma showed that CSC‐derived exosomes promoted EMT in renal carcinoma cells by transferring miR‐19b‐3p.^18^ As one of the most important and essential components of the tumour microenvironment, whether lung CSC‐derived exosomes carrying special microRNA involves in the pro‐metastatic phenotype of lung cancer cells and its underlying molecular mechanism remains unclear.

In the present study, we identified and enriched lung CSCs from parental A549 cells and explored the functional role of lung CSC‐derived exosomes in regulation of the pro‐metastatic phenotype of lung cancer cells. We also investigated the underlying molecular mechanism.

## 2 MATERIALS AND METHODS

### 2.1 Cell culture

Lung cancer cell lines including A549, NCI‐H1703 and human normal lung epithelial cell line BEAS‐2B were obtained from the Shanghai Cell Bank of the Chinese Academy of Sciences (Shanghai, China). The A549 and BEAS‐2B cell lines were grown in Dulbecco's modified Eagle medium (DMEM) (Hyclone). The NCI‐H1703 cell line was cultured in RPMI‐1640 medium. All media were supplemented with 10% foetal bovine serum (FBS), streptomycin (100 μg/mL) and penicillin (100 U/mL). Cells were grown at 37°C in a humidified 5% CO_2_ atmosphere.

To generate suspended and stem‐like sphere‐growing cells, A549 cells were dissociated into single cells and seeded onto 6‐well ultra‐low attachment plates (Corning) at a concentration of 1 × 10^4^ cells/well. Cells were incubated in DMEM/F12 (Gibco) supplemented with recombinant human epidermal growth factor (rhEGF, 10 ng/mL; Sigma), basic fibroblast growth factor (bFGF, 10 ng/mL; Sigma) and insulin (4 U/I; Sigma) for 12 days. After primary tumour spheres reached approximately 50‐100 μm/sphere, the spheres were dissociated with Accutase (Invitrogen). The single cells obtained were cultured for another 12 days until secondary spheres had formed. After 5 passages, the spheres were collected, and characterization experiments were performed.

### 2.2 Cell transfection with miR‐210‐3p mimic, miR‐210‐3p inhibitor, FGFRL1 siRNA or pcDNA3.1‐FGFRL1

MiR‐210‐3p mimic, miR‐210‐3p inhibitor and corresponding negative controls (miR‐NC and miR‐inhibitor NC, respectively) were purchased from GenePharma. A small interfering RNA (siRNA) targeting human FGFRL1 mRNA, its negative control siRNA and pcDNA3.1‐FGFRL1 as well as pcDNA3.1 blank vector were also purchased from GenePharma. Lipofectamine 3000 reagent (Invitrogen) was used according to the manufacturer's protocol under serum‐free conditions. After 6‐12 h of transfection, the liquid was abandoned and replaced with fresh medium, and cells were incubated for another 24 or 48 h. Cells were then collected, and transfection efficiency was verified by quantitative real‐time polymerase chain reaction (qPCR) analysis or Western blot.

### 2.3 Tumour sphere formation

Single cells derived from tumour spheres or parental A549 cells were seeded in 96‐well ultra‐low plates at a density of 50 or 100 cells per well, respectively. After 12 days of non‐adhesive culture, the number of spheres with >50 µm/sphere was counted, and representative morphology was captured under light microscopy (Leica). Sphere‐forming efficiency (SFE) was calculated as the number of spheres formed divided by the initial number of single cells plated and expressed as a percentage.

### 2.4 Quantitative real‐time PCR

Total RNA from cells or exosomes was extracted using TRIzol reagent (Invitrogen). The primer sequences for ALDH1, Sox2, Nanog, CD133, BDNF, E2F3, FGFRL1, KCMF1, NDUFA4, GAPDH, miR‐210‐3p and U6 were designed and synthesized by GenePharma. The primer sequences are listed in Table 1. RNAs were reverse‐transcribed with PrimeScript Reverse Transcriptase (Takara) or microRNA first‐strand cDNA synthesis (Sangon Biotech); qPCR analysis was performed with the SYBR Green Detection Kit (Takara) or the MicroRNA qPCR Kit (SYBR Green Method) (Sangon Biotech). All processes were performed according to the manufacturer's instructions. GAPDH and U6 were used as endogenous controls.

**Table 1:** Primer sequences

Primer	Sequence (5’‐3’)
CD133	Forward 5’‐TCTTGACCGACTGAGACCCAAC‐3’ Reverse 5’‐ACTTGATGGATGCACCAAGCAC‐3’
ALDH1	Forward 5’‐TTGCTATGGCGTGGTAAGT‐3’ Reverse 5’‐GACTTACCTGTCCTACTCA‐3’
Nanog	Forward 5’‐CTCGCTGATTAGGCTCCAAC‐3’ Reverse 5’‐TTCAGTCTGGACACTGGCTG‐3’
Sox2	Forward 5’‐CGATGCCGACAAGAAAACTT‐3’ Reverse 5’‐CAAACTTCCTGCAAAGCTCC‐3’
BDNF	Forward 5’‐CCTCTCAGCCTCCAGCGTTG‐3’ Reverse 5’‐TGCTCTTGCTCACTCACACT‐3’
E2F3	Forward 5’‐AAGAAATTAGATGAACTGATCCAAAGC‐3’ Reverse 5’‐TAACATAAGCTAACCTTTGATTCTCTGA‐3’
FGFRL1	Forward 5’‐TGTGAACACAACGGTGGACT‐3’ Reverse 5’‐GGGCAACACCACAAACTTCT‐3’
KCMF1	Forward 5’‐TGAGACGTTCTGCAGGAGGAC‐3’ Reverse 5’‐TTGTGTTAGTACGCCGGGTTG‐3’
NDUFA4	Forward 5’‐AAGCATCCGAGCTTGATCCC‐3’ Reverse 5’‐TGGGACCCAGTTTGTTCCAG‐3’
GAPDH	Forward 5’‐GACCTGACCTGCCGTCTA‐3’ Reverse 5’‐AGGAGTGGGTGTCGCTGT‐3’
miR‐210‐3p	Forward 5’‐GGACTAGGTCATCTGCATACG‐3’ Reverse 5’‐CCGAATGATGTCGCTTACGAC‐3’
U6	Forward 5’‐CTCGCTTCGGCAGCACA‐3’ Reverse 5’‐AACGCTTCACGAATTTG‐3’

### 2.5 Immunofluorescent staining assay

Tumour spheres were collected and fixed with 4% paraformaldehyde for 10 min before they were embedded in low‐melting point agarose for 30 min at 4°C. The coagulated agarose was embedded in OCT compound (Tissue Tek). Frozen blocks were cut into section of 5‐µm thickness. A549 cells were plated onto Matrigel‐coated glass coverslips before fixation with 4% paraformaldehyde for 10 min at room temperature, followed by washes in phosphate‐buffered saline (PBS). All cells were permeabilized with 0.5% Triton X‐100 for 15 min at room temperature and blocked in 5% BSA for 30 min before incubation with CD133 and ALDH1 (1:300, Beijing Biosynthesis Biotechnology Co., LTD). Sections were incubated in primary antibodies overnight at 4℃ and then incubated with fluorescence‐tagged mouse or rabbit secondary antibodies. DAPI (Sigma‐Aldrich) was used for nuclear staining. Fluorescence images were visualized with a fluorescence microscope (Leica).

### 2.6 Western blot analysis

Cells or exosomes were treated with RIPA lysis buffer supplemented with protease inhibitor cocktail (Beyotime Biotechnology). Samples containing 20 μg protein were separated and transferred to PVDF membranes, and then blocked in 5% non‐fat milk at room temperature for 1 h. The PVDF membranes were incubated with primary antibodies for rabbit anti‐CD133, anti‐Nanog, anti‐Sox2, anti‐ALDH1, anti‐FGFRL1 and anti‐β‐actin (1:500, Beijing Biosynthesis Biotechnology Co., LTD); rabbit anti‐CD63, rabbit anti‐TSG101 and rabbit anti‐CD81 (1:500, Abcam); and rabbit anti‐E‐cadherin, anti‐N‐cadherin, anti‐vimentin, anti‐MMP‐9 and anti‐MMP‐1 (1:1000, Cell Signaling Technology, Inc) at 4°C overnight. Antibody recognition was performed with peroxidase‐conjugated goat anti‐rabbit IgG (H + L) secondary antibody (Zhongshan Goldenbridge Biotechnology Co., LTD), which was used at a 1:5000 dilution. Protein levels were detected with chemiluminescent HRP substrate and a Western blot analysis system (Universal Hood II, Bio‐Rad).

### 2.7 Tumorigenicity in immunodeficient mice

To evaluate tumorigenicity, approximately 10^3^, 10^4^, 10^5^ and 10^6^ of parental A549 cells and 10^2^, 10^3^, 10^4^ and 10^5^ of tumour sphere‐derived cells were suspended in 100 μl PBS and then inoculated into the armpits of nude mice (five weeks old, BALB/C‐nu/nu, female, n = 3). Five weeks after inoculation, mice were killed, and tumour tissues were collected. Animals were purchased from Hua Fu Kang Bioscience Incorporated Company (Beijing, China) and maintained under standard conditions, according to the guidelines of the Institutional Animal Care and Use Committee of Sichuan University. All animal experiments were performed in compliance with the authenticated animal protocols of the Institutional Animal Care and Use Committee of Sichuan University.

### 2.8 Exosome isolation and quantification

For lung cancer cell culture, the medium was changed to RMPI‐1640 or DMEM containing 10% exosome‐free FBS (System Biosciences) for 48 h, until cells reached 90% confluency and were subsequently collected. For tumour spheres, medium was collected from supernatant with approximately 2 × 10^6^ cells/mL. After using a 0.22‐μm filter (Millipore) to remove cellular debris, the medium obtained was ultra centrifuged at 120 000 × *g*, at 4°C for 60 min, to pellet the exosomes. The supernatant was discarded. The pellet was washed with PBS, ultracentrifuged and finally resuspended again in ice‐cold PBS for further analysis. The concentration of exosomes was determined by Pierce bicinchoninic acid (BCA) protein assay, according to the manufacturer's instructions.

### 2.9 Transmission electron microscopy

The purified exosomes were prepared by mixing with an equal volume of 4% paraformaldehyde and deposited on Formvar carbon‐coated copper grids. Grids were stained with 2% uranyl acetate for 15 min, air‐dried and then observed by transmission electron microscopy (TEM; FEI Tecnai 20; Philips).

### 2.10 Nanoparticle tracking analysis

To measure the size of the purified exosomes, they were resuspended in PBS. NanoSight NS300 (Malvern Instruments) nanoparticle tracking analysis (NTA) software was then used to visualize and measure particle size.

### 2.11 PKH26 labelling of exosomes

To observe the interaction between exosomes and lung cancer cells, exosomes were labelled with PKH26 (a membrane fluorescence dye that is red in colour) (Sigma‐Aldrich). After co‐incubation with PKH26‐labelled exosomes for 6 h, lung cancer cells were counterstained with DAPI. Staining patterns were visualized with an inverted fluorescence microscope linked to a camera (Leica).

### 2.12 Detection of miR‐210‐3p transfer

Tumour spheres were transiently transfected with fluorescein amidite (FAM)‐labelled miR‐210‐3p mimic (GenePharma) with Lipofectamine 3000. After transfection for 48 h, the culture medium was collected to extract exosomes, as described above. Exosomes isolated from the culture medium of tumour spheres transfected with FAM‐labelled miR‐210‐3p were labelled with Dil (Invitrogen) and then added to the lung cancer cell culture. After incubation for 24 h, cells were counterstained with DAPI. Images were visualized using an inverted fluorescence microscope linked to a camera (Leica, Japan).

### 2.13 MTT assay

Lung cancer cells were seeded in 100 μL of medium/well (2 × 10^3^ cells per well) in 96‐well plates. After co‐incubation with various concentrations of exosomes (0, 10, 20, 40 or 80 μg/mL) for 1, 2, 3, 4 or 5 days, 10 μL of MTT was added to each well. Cells were incubated for 4 h. Then, 100 μL of 10% SDS/0.01 N HCL was added to each well. Plates were incubated at 37°C overnight to dissolve the formazan. Absorbance was measured at 450 nm.

### 2.14 Cell migration assay

A wound‐healing assay was performed to assess migratory ability under various conditions. Briefly, cells were seeded in 6‐well plates to create a confluent monolayer. A scratch wound was made with a sterile pipette tip, after which plates were incubated for 24 or 48 h with or without exosomes. Images were captured under a camera equipped with a light microscope (Leica).

### 2.15 Transwell assay

Cell migration and invasion were evaluated using Transwell chambers (8‐μm pore size; Millipore, Massachusetts, USA) with or without Matrigel (BD Biosciences) matrix. In brief, cells were added to the upper chamber; the bottom chamber was filled with complete medium. After 48 h, the cells on the top surface of the membrane were mechanically removed using a cotton swab. Cells that had migrated towards or penetrated the membrane were stained with a 4 g/L crystal violet solution. The number of cells was counted under an inverted light microscope linked to a camera (Leica).

### 2.16 Dual‐luciferase reporter assay

The amplified FGFRL1‐3’‐UTR‐WT and corresponding FGFRL1‐3’‐UTR‐MUT were cloned into pGL3 luciferase vector (Promega Corporation). Lung cancer cells were seeded onto 96‐well plates over a 24‐h period. Lung cancer cells were transfected with FGFRL1‐3’‐UTR‐WT and miR‐210‐3p mimic or FGFRL1‐3’‐UTR‐MUT and miR‐210‐3p mimic using Lipofectamine 3000. After 48 h, cells were harvested, and the Dual Luciferase Reporter Assay Kit (Promega Corporation) was used to measure relative differences in luciferase activity.

### 2.17 Statistical analysis

Differences were examined using Student's *t* test or one‐way analysis of variance. The data are presented as means ± SD. Values of *P* < .05 were considered statistically significant. All statistical analyses were performed using SPSS version 17.0 software (IBM).

## 3 RESULTS

### 3.1 Identifying stemness phenotype in A549 cell‐derived tumour spheres

**Figure 1:**
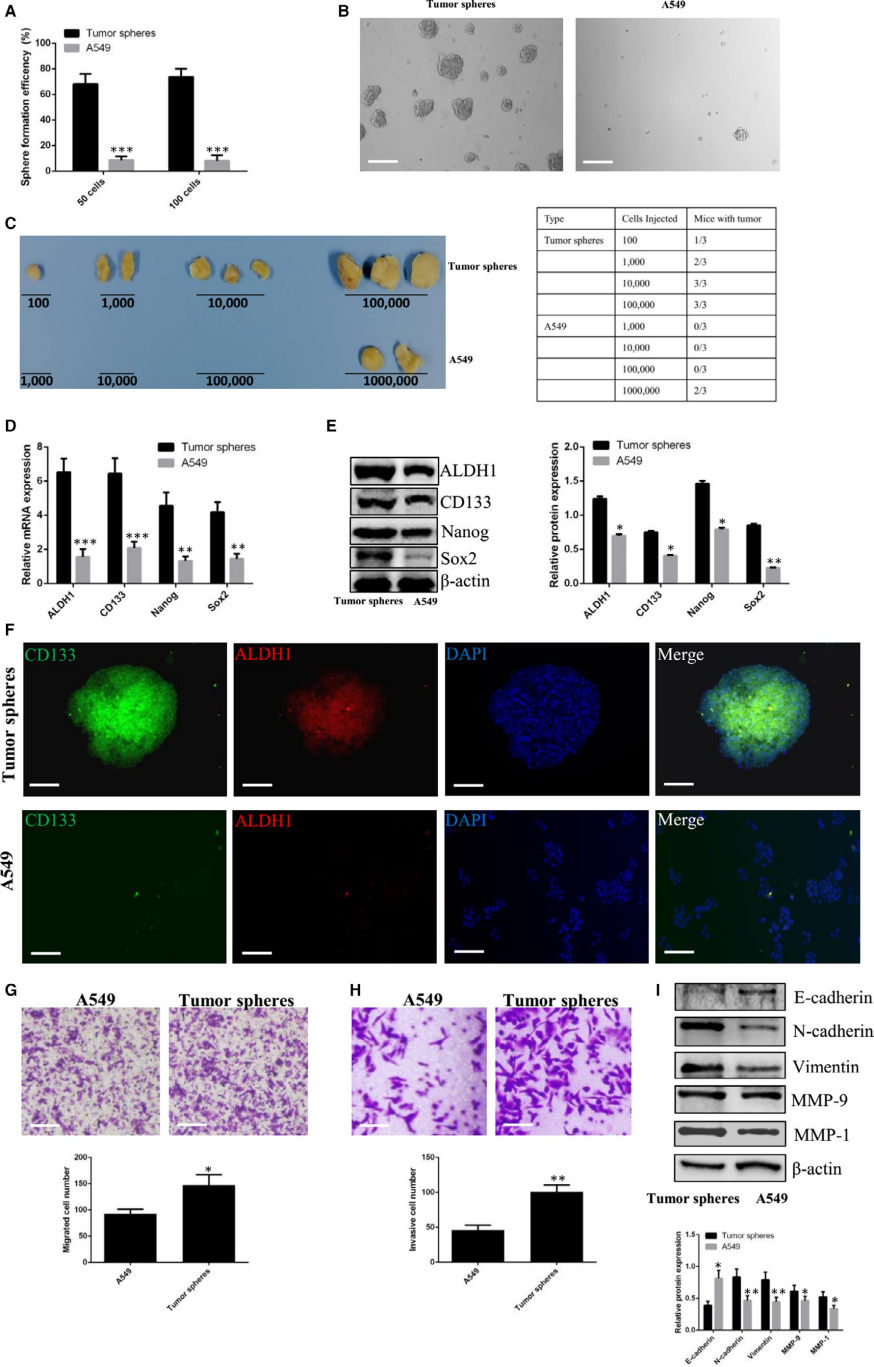
Identification of stemness phenotype in tumour spheres derived from parental A549 cells. A, The diagram shows the SFE of tumour sphere‐derived cells and parental A549 cells, respectively. B, Representative images of sphere formation from tumour sphere‐derived cells and parental A549 cells cultured in non‐adhesive conditions for 12 days. Scale bars: 100 µm. C, Tumorigenesis of tumour sphere‐derived cells or parental A549 cells in nude mice (n = 3). D, qPCR analysis of ALDH1, CD133, Nanog and Sox2. E, Western blot analysis of ALDH1, CD133, Nanog and Sox2. F, Immunofluorescent staining of tumour spheres and parental A549 cells. Scale bars: 100 μm. G, Transwell migration of tumour sphere‐derived cells and parental A549 cells. Scale bars: 200 μm. H, Transwell invasion of tumour sphere‐derived cells and parental A549 cells. Scale bars: 100 μm. I, Western blot analysis of E‐cadherin, N‐cadherin, vimentin, MMP‐9 and MMP‐1. Tumour spheres vs. A549 cells; * *P* < .05. ** *P* < .01. *** *P* < .001. Data are mean ± SD from three independent experiments performed in triplicate

Self‐renewal ability is the most important characteristics of CSCs. As shown in Figure 1A, through limiting dilution analysis, we demonstrated that the SFE of tumour sphere‐derived cells was significantly increased, compared to that of parental A549 cells. Indeed, single cells derived from tumour spheres were able to form new spheres, whereas parental A549 cells had no such effect. Most A549 cells present in the non‐adhesive culture appeared to be dead (Figure 1B). When we evaluated tumorigenesis in vivo, we found that inoculation with 10^2^, 10^3^, 10^4^ or 10^5^ tumour spheres generated tumours in 1/3, 2/3, 3/3 and 3/3 of inoculations, respectively. Inoculation with 10^3^, 10^4^, 10^5^ and 10^6^ of parental A549 cells generated tumours in 0/3, 0/3, 0/3 and 2/3 of inoculations (Figure 1C). Stemness‐associated markers including ALDH1, Sox2, Nanog and CD133 were closely involved with maintenance of the stemness phenotype in lung CSCs. Through qPCR and Western blot analysis, we verified that the expression levels of these markers were significantly higher in tumour spheres than in parental A549 cells (Figure 1D‐E). Immunofluorescent staining analysis further revealed that the intensity of ALDH1 and CD133 fluorescence was higher in tumour spheres, compared to parental A549 cells (Figure 1F). CSCs acquire enhanced metastatic ability during the EMT. The results of the Transwell assays performed in this study showed that the number of migrated or invaded cells derived from tumour spheres was greatly increased, compared to the number of migrated or invaded cells derived from parental A549 cells (Figure 1G‐H). Western blot analysis of tumour spheres further showed that the expression of EMT‐associated proteins including N‐cadherin, vimentin, MMP‐9 and MMP‐1 was up‐regulated, whereas E‐cadherin expression was down‐regulated (Figure 1I). These results suggest that tumour spheres derived from parental A549 cells expressed the stemness phenotype. These cells appeared to have dedifferentiated into lung CSCs.

### 3.2 Lung cancer cells internalized lung CSC‐derived exosomes

**Figure 2:**
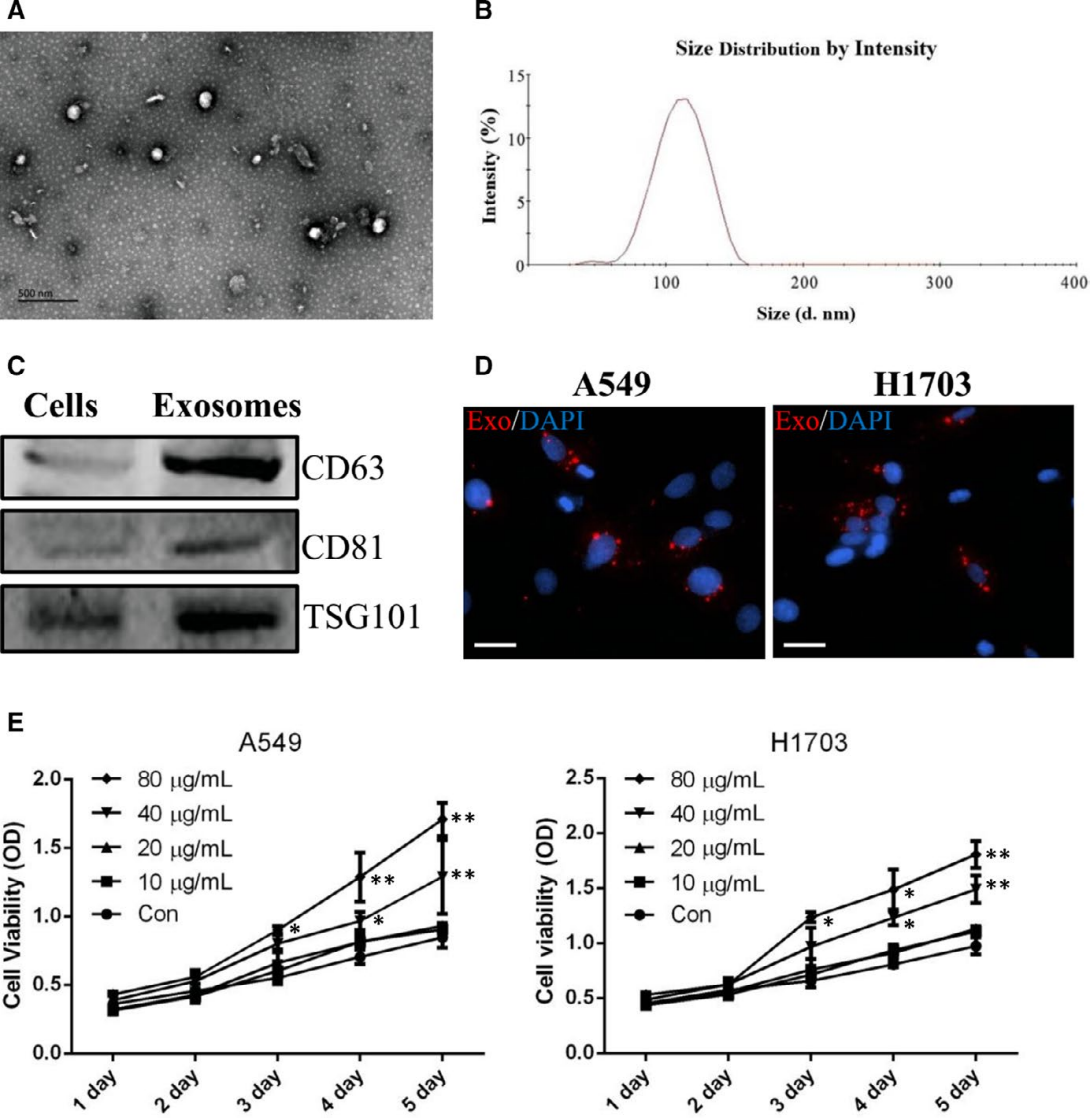
Characterization of exosomes purified from lung CSCs. A, Representative transmission electron micrographic image of exosomes. Scale bar: 500 nm. B, Nanoparticle tracking showing exosome diameter. C, Western blot analysis of CD63, CD81 and TSG101. D, Representative images of PKH26‐labelled exosomes co‐cultured with A549 and NCI‐H1703 cells. Scale bars: 100 μm. E, MTT assay analysing cell viability of A549 and NCI‐H1703 cells after treatment with lung CSC‐derived exosomes. Control vs. 10, 20, 40 or 80 μg/mL exosomes, respectively. * *P* < .05. ** *P* < .01. Data are mean ± SD from three independent experiments performed in triplicate

To investigate the effects of exosomes in lung cancer cells, we purified exosomes from lung CSCs. As shown in Figure 2, the characteristics of exosomes were verified through TEM, NTA and Western blot analysis. TEM observation revealed that most vesicles had a typical cup‐shaped morphology (Figure 2A). NTA analysis showed that the average diameter of exosomes ranged from 30 to 150 nm (Figure 2B). Western blot analysis further demonstrated that these exosomes expressed special markers including CD63, TSG101 and CD81 (Figure 2C). Next, we labelled these exosomes with PKH26 to observe their interactions with lung cancer cells. As shown in Figure 2D, lung cancer cells internalized lung CSC‐derived exosomes, indicating that CSC‐derived exosomes may regulate lung cancer cells. Interestingly, the results of MTT analysis showed that when lung cancer cells were co‐incubated with lung CSC‐derived exosomes for more than 2 days, cell proliferation increased (Figure 2E).

### 3.3 Lung CSC‐derived exosomes contribute to a pro‐metastatic phenotype in lung cancer cells

**Figure 3:**
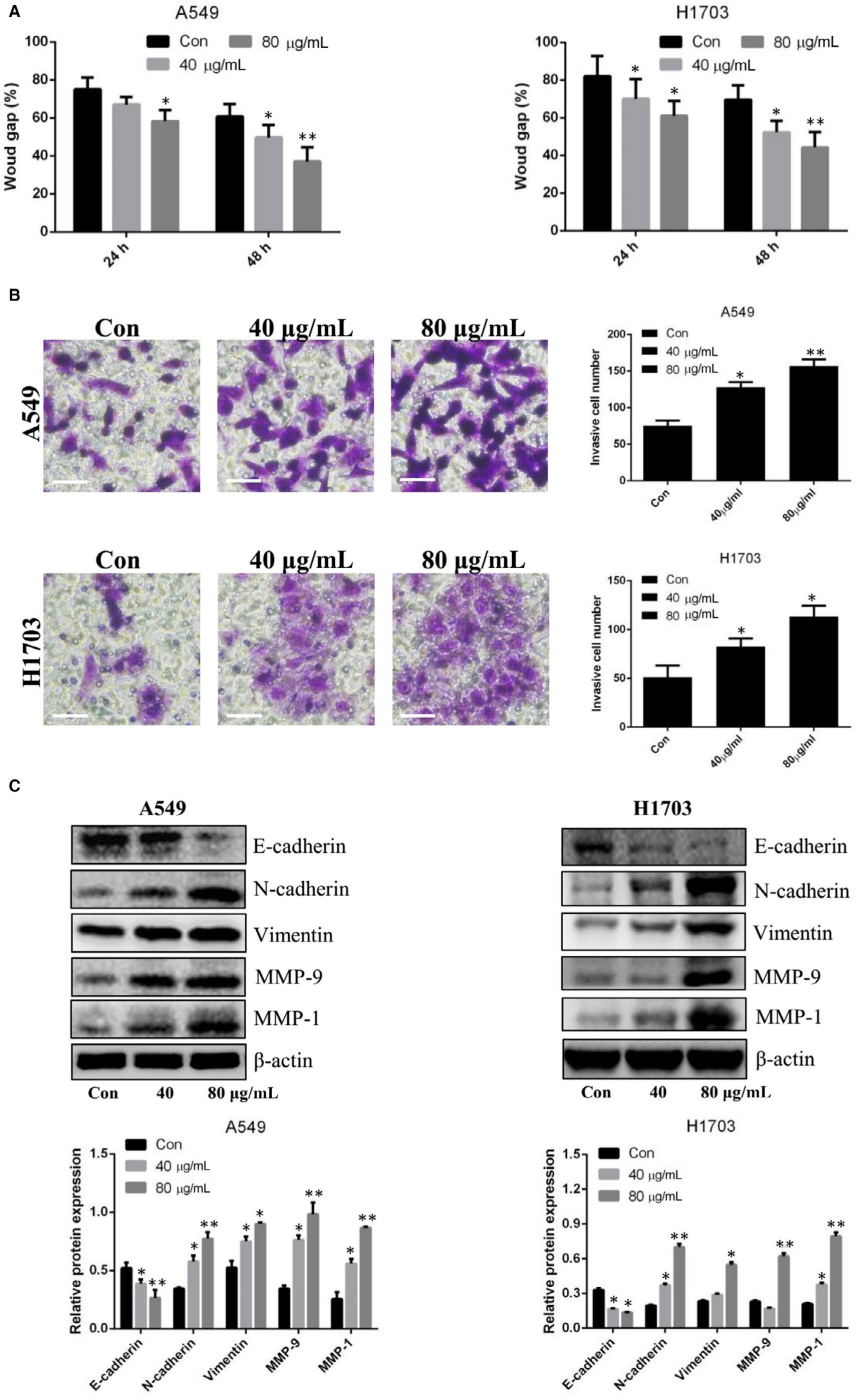
Exosomes exert pro‐metastatic effects in lung cancer cells. A, The diagram shows the migratory ability of A549 and NCI‐H1703 cells after treatment with lung CSC‐derived exosomes. B, Transwell invasion of A549 and NCI‐H1703 cells after treatment with lung CSC‐derived exosomes. Scale bars: 100 μm. C, Western blot analysis for E‐cadherin, N‐cadherin, vimentin, MMP‐9 and MMP‐1. Control vs. 40 or 80 μg/mL exosomes, respectively. *P < .05. **P < .01. Data are mean ± SD from three independent experiments performed in triplicate

To clarify the possibility of a pro‐metastatic role for exosomes, lung cancer cells were co‐incubated with lung CSC‐derived exosomes. Our results showed that these exosomes enhanced the migration and invasion of lung cancer cells in a dose‐dependent manner (Figure 3A,B and Figure S1). Moreover, through Western blot analysis of lung cancer cells treated with lung CSC‐derived exosomes, we demonstrated that expression levels of N‐cadherin, vimentin, MMP‐9 and MMP‐1 were up‐regulated, whereas E‐cadherin expression was down‐regulated (Figure 3C).

### 3.4 Exosomal miR‐210‐3p regulated the metastatic potential of lung cancer cells

**Figure 4:**
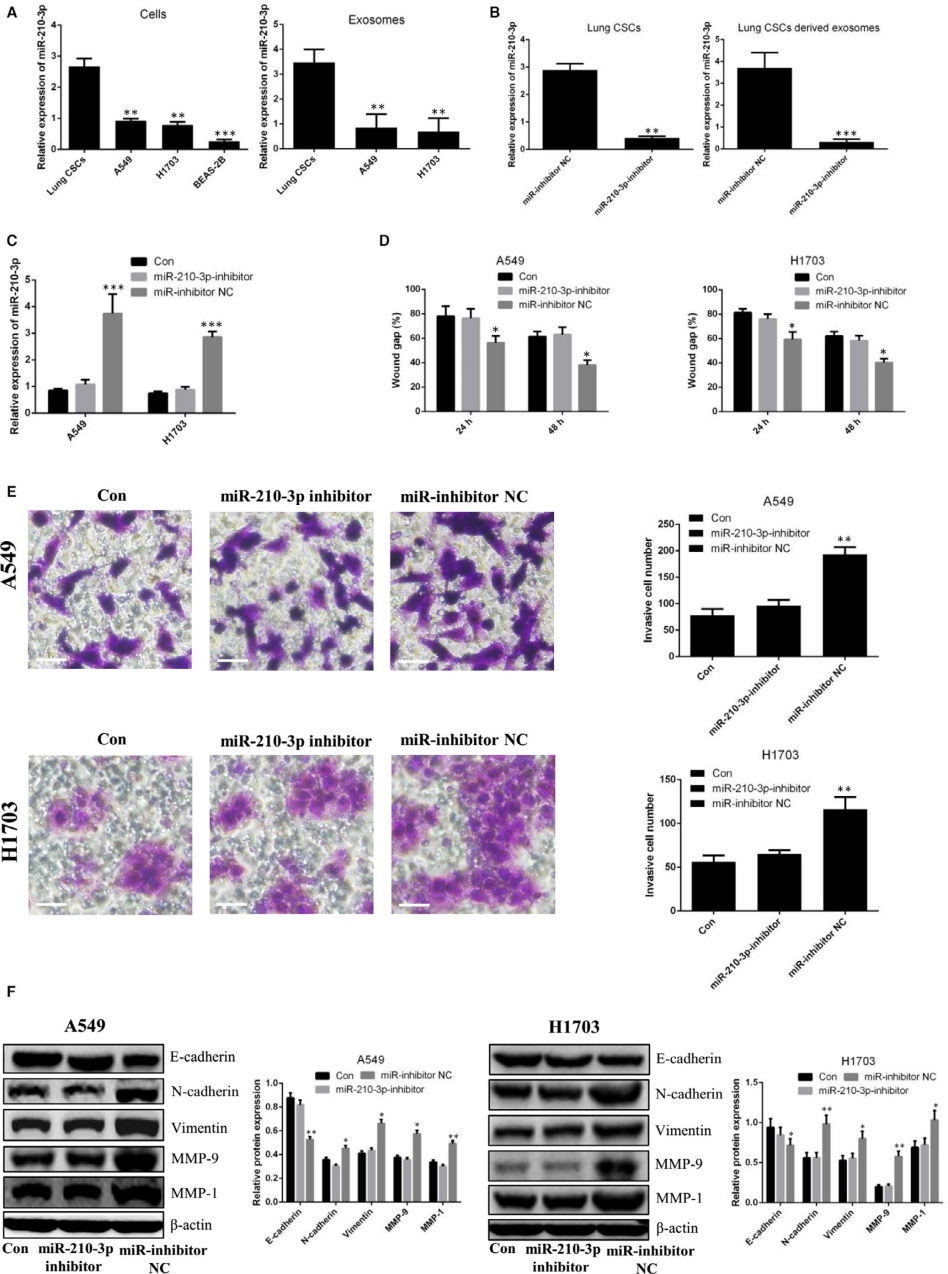
Exosomal miR‐210‐3p regulates the metastatic potential of lung cancer cells derived from lung CSCs transfected with miR‐210‐3p inhibitor. A, qPCR analysis of miR‐210‐3p levels in lung CSCs, A549 cells, NCI‐H1703 cells, BEAS‐2B cells and their derived exosomes. Lung CSCs/exosomes vs. A549, NCI‐H1703 or BEAS‐2B/exosomes, respectively. B, qPCR analysis of miR‐210‐3p levels in lung CSCs and their derived exosomes after transfection with miR‐210‐3p inhibitor. miR‐inhibitor NC vs. miR‐210‐3p inhibitor. C, qPCR analysis of miR‐210‐3p levels in A549 and NCI‐H1703 cells after co‐incubation with exosomes (80 μg/mL) derived from lung CSCs transfected with miR‐210‐3p inhibitor or miR‐inhibitor NC. D, The diagram shows the migratory ability of A549 and NCI‐H1703 cells after co‐incubation with exosomes (80 μg/mL) derived from lung CSCs transfected with miR‐210‐3p inhibitor or miR‐inhibitor NC. E, Transwell invasion of A549 and NCI‐H1703 cells after co‐incubation with exosomes (80 μg/mL) from lung CSCs transfected with miR‐210‐3p inhibitor or miR‐inhibitor NC. Scale bars: 100 μm. F, Western blot analysis of E‐cadherin, N‐cadherin, vimentin, MMP‐9 and MMP‐1. Control vs. miR‐inhibitor NC, miR‐210‐3p inhibitor; *P < .05. **P < .01. ***P < .001. Data are mean ± SD from three independent experiments performed in triplicate

**Figure 5:**
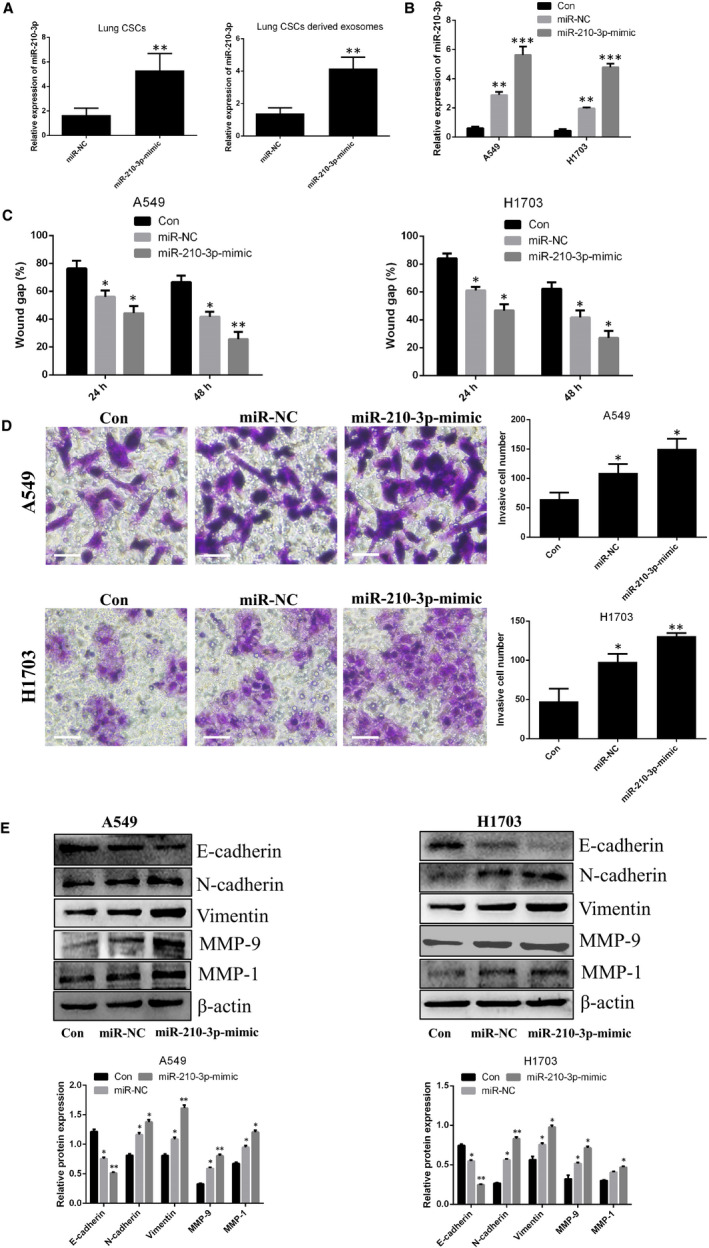
Exosomal miR‐210‐3p regulates the metastatic potential of lung cancer cells derived from lung CSCs transfected with miR‐210‐3p mimic. A, qPCR analysis of miR‐210‐3p levels in lung CSCs and their derived exosomes after transfection of lung CSCs with miR‐210‐3p mimic (miR‐NC vs. miR‐210‐3p mimic). B, qPCR analysis of miR‐210‐3p levels in A549 and NCI‐H1703 cells after co‐incubation with exosomes (80 μg/mL) derived from lung CSCs transfected with miR‐210‐3p mimic or miR‐NC. C, The diagram shows the migratory ability of A549 and NCI‐H1703 cells after co‐incubation with exosomes (80 μg/mL) derived from lung CSCs transfected with miR‐210‐3p mimic or miR‐NC. D, Transwell invasion of A549 and NCI‐H1703 cells after co‐incubation with exosomes (80 μg/mL) derived from lung CSCs transfected with miR‐210‐3p mimic or miR‐NC. Scale bars: 100 μm. E, Western blot analysis for E‐cadherin, N‐cadherin, vimentin, MMP‐9 and MMP‐1. Control vs. miR‐NC, miR‐210‐3p mimic; *P < .05. ** *P* < .01. Data are mean ± SD from three independent experiments performed in triplicate

Recent reports indicate that miR‐210‐3p plays a central role in the metastasis of prostate cancer and renal cell carcinoma.^19^, ^20^ More importantly, mesenchymal stem cell‐derived exosomes promoted lung cancer metastasis via the transfer of miR‐210‐3p.^21^ In this study, we demonstrated that lung CSC‐derived exosomes promote lung cancer metastasis. Based on the results obtained, we speculate that exosomes may be associated with the transfer of miR‐210‐3p. We therefore analysed miR‐210‐3p levels in cells and their derived exosomes. As shown in Figure 4A, miR‐210‐3p levels were significantly higher in lung CSCs, compared with lung cancer cells. Similarly, miR‐210‐3p levels were increased in lung CSC‐derived exosomes, compared with lung cancer cell‐derived exosomes. After lung CSCs were transfected with miR‐210‐3p inhibitor, miR‐210‐3p levels were clearly decreased in lung CSCs and their derived exosomes, compared to lung CSCs transfected with miR‐inhibitor NC and derived exosomes (Figure 4B). After lung cancer cells were co‐incubated with miR‐210‐3p inhibitor‐transfected lung CSC‐derived exosomes, there was no change in the level of miR‐210‐3p. However, there was an increase in miR‐210‐3p expression in lung cancer cells transfected with miR‐inhibitor NC, compared to the control group (Figure 4C). Moreover, no change in migration, invasion or expression of EMT‐associated proteins was observed in lung cancer cells incubated with miR‐210‐3p inhibitor‐transfected lung CSC‐derived exosomes (Figure 4D‐F). In contrast, the exosomes derived from lung CSCs transfected with miR‐210‐3p mimic enhanced the metastatic abilities of lung cancer cells (Figure 5A‐E). To confirm the direct transfer of miR‐210‐3p from lung CSC‐derived exosomes to lung cancer cells, we transfected lung CSCs with FAM‐miR‐210‐3p mimic and then labelled secreted exosomes containing FAM‐miR‐210‐3p with Dil. Under fluorescence microscopy, red and green signals were detected in the cytoplasm of lung cancer cells exposed to these Dil‐labelled exosomes (Figure S2A). This result was in accordance with our finding that miR‐210‐3p expression was significantly increased in lung cancer cells treated with exosomes derived from miR‐210‐3p mimic‐transfected lung CSCs, compared to lung cancer cells treated with exosomes derived from the untreated group (Figure 5B).

After verifying the pro‐proliferative effect of exosomes on lung cancer cells, we investigated the role of miR‐210‐3p in this process. As shown in Figure S2B, exosomes derived from lung CSCs transfected with miR‐210‐3p mimic or miR‐210‐3p inhibitor promoted the proliferation of lung cancer cells, compared to levels of proliferation observed in control lung cancer cells. This pro‐proliferative effect was more readily apparent in the cells treated with exosomes from lung CSCs transfected with miR‐210‐3p mimic.

### 3.5 FGFRL1 may be a target of miR‐210‐3p in lung cancer cells

**Figure 6:**
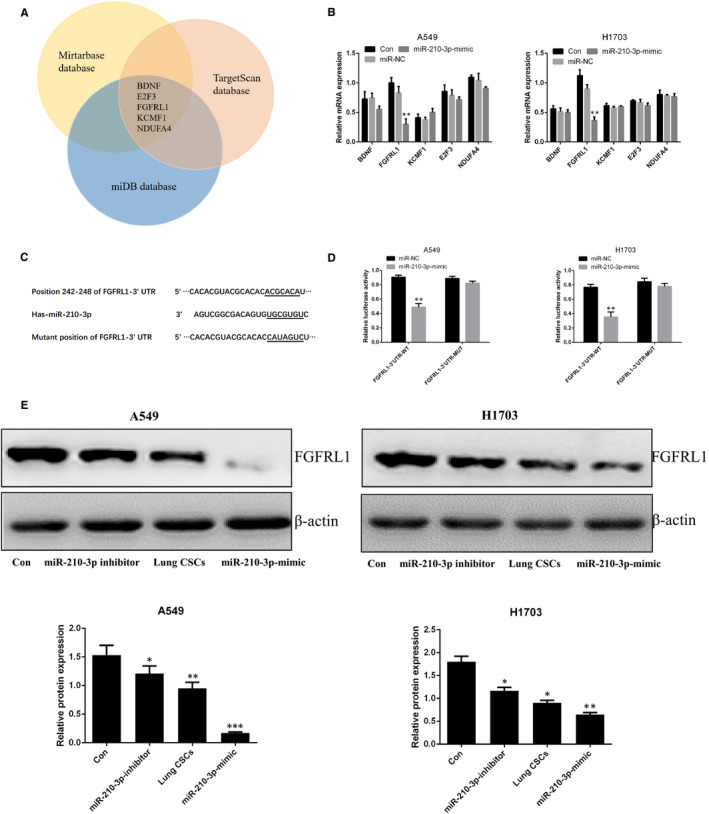
miR‐210‐3p promotes a pro‐metastatic phenotype by targeting FGFRL1. A, TargetScan, miRTarBase and miDB software analyses of potential targets of miR‐210‐3p. B, qPCR analysis of BDNF, E2F3, FGFRL1, KCMF1 and NDUFA4. Control vs. miR‐210‐3p mimic or miR‐NC, respectively. C, Sequence alignment of the binding sites on the 3’UTR of miR‐210‐3p and FGFRL1. D, Luciferase activity in A549 and NCI‐H1703 cells after transfection. miR‐NC vs. miR‐210‐3p mimic. E, Western blot analysis of FGFRL1. *P < .05. **P < .01. Data are mean ± SD from three independent experiments performed in triplicate

**Figure 7:**
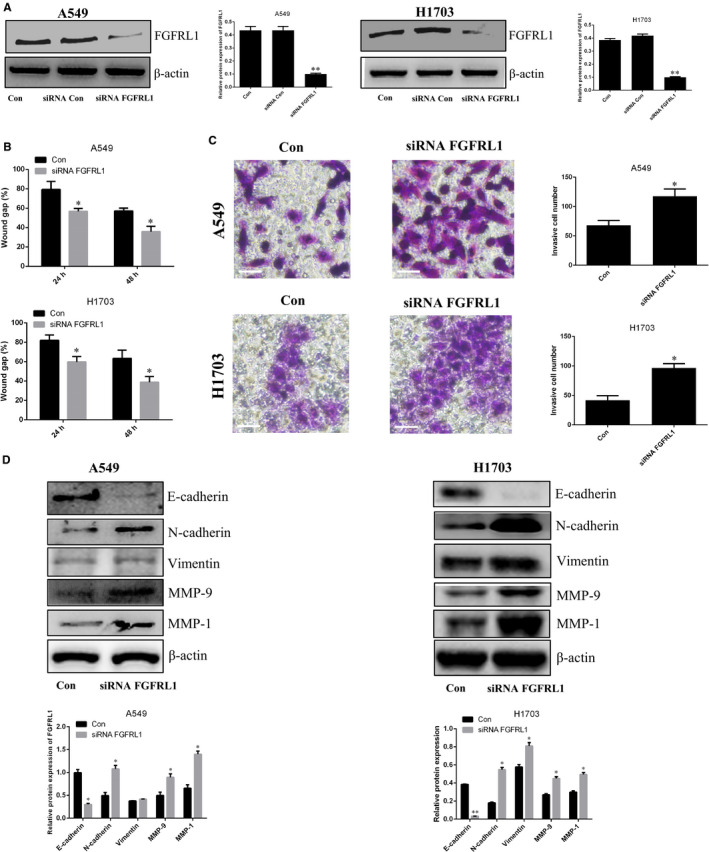
Silencing FGFRL1 promotes metastasis in lung cancer cells. A, Western blot analysis of FGFRL1. B, The diagram shows the migratory ability of A549 and NCI‐H1703 cells after transfection with siRNA‐FGFRL1. C, Transwell invasion of A549 and NCI‐H1703 cells after transfection with siRNA‐FGFRL1. Scale bars: 100 μm. D, Western blot analysis of E‐cadherin, N‐cadherin, vimentin, MMP‐9 and MMP‐1. Control vs. siRNA‐FGFRL1. *P < .05. **P < .01. Data are mean ± SD from three independent experiments performed in triplicateAUTHOR: Figure 5 has been saved at a low resolution of 190 dpi. Please resupply at 600 dpi. Check required artwork specifications at https://authorservices.wiley.com/asset/photos/electronic_artwork_guidelines.pdf

To further verify the molecular mechanism by which miR‐210‐3p promotes a pro‐metastatic phenotype in lung cancer cells, three bioinformatics software programs (TargetScan, miRTarBase and miDB) were used to identify possible potential downstream targets of miR‐210‐3p. As shown in Figure 6A, five potential genes (BDNF, E2F3, FGFRL1, KCMF1 and NDUFA4) were screened. The results of qPCR analysis demonstrated that, among these genes, FGFRL1 was expressed at significantly decreased levels in lung cancer cells transfected with miR‐210‐3p mimic (Figure 6B). The results of the dual‐luciferase reporter assay verified that FGFRL1 may be a target of miR‐210‐3p (Figure 6C,D). In order to clarify the relationship between lung CSC‐derived exosomal miR‐210‐3p and FGFRL1, we performed Western blot analysis to measure the expression level of FGFRL1 in lung cancer cells after co‐incubation with lung CSC‐derived exosomes. As shown in Figure 6E, FGFRL1 levels were both decreased in lung cancer cells incubated with miR‐210‐3p mimic or miR‐210‐3p inhibitor‐transfected lung CSC‐derived exosomes, compared with control levels; the decreased levels were more apparent in lung cancer cells after incubation with exosomes from lung CSCs transfected with miR‐210‐3p mimic. When lung cancer cells in which FGFRL1 had been silenced with siRNA were compared with lung cancer cells, migratory and invasive potential were enhanced; expression levels of N‐cadherin, vimentin, MMP‐9 and MMP‐1 increased; and E‐cadherin expression decreased (Figure 7A‐D). However, metastatic ability decreased in lung cancer cells overexpressing FGFRL1 (Figure S3A‐D).

## 4 DISCUSSION

During recent years, new therapeutic agents have emerged for lung cancer patients. Updated therapeutic strategies include the use of third‐generation EGFR/TKI or PD‐1/PD‐L1 checkpoint inhibitors. However, the overall survival of patients with advanced lung cancer remains low, and the clinical prognosis is poor.^22^ Metastasis is the most common cause of death in this patient population. The suppression of lung cancer metastasis may be a promising and feasible therapeutic strategy for improving survival among lung cancer patients.

Lung CSCs play an important role in tumour metastasis and recurrence. In this study, the tumour spheres derived from parental A549 cells overexpressed stemness‐associated markers including CD133, Nanog, Sox2 and ALDH1, which are associated with the lung CSC phenotype.^23^, ^24^ The gold standard for the differentiation of CSCs from parental cancer cells is the capacity for self‐renewal.^25^ By analysing SFE in vitro, we verified that SFE was significantly increased in tumour spheres, compared to parental A549 cells. Another characteristic associated with the capacity for self‐renewal is the enhanced tumorigenesis of lung CSCs in vivo. One previous study of a mouse model indicated that inoculation with only 10^2^ lung CSCs resulted in xenograft formation.^26^ In this study, we demonstrated that inoculation with 10^2^ tumour spheres generated xenografts, whereas there was no xenograft formation in mice inoculated subcutaneously with 10^5^ parental A549 cells. These findings provide further support for the potent self‐renewal of tumour spheres. EMT has been confirmed to be an essential step for metastasis. E‐cadherin, N‐cadherin and vimentin have been identified as the main proteins associated with cell migration.^27^ MMP‐9 and MMP‐1 are proteolytic enzymes that facilitate cell invasion by degrading extracellular matrix, which leads to metastasis.^28^ Previous reports indicated that lung CSCs were endowed with EMT characteristics and increased risk for metastasis.^29^ Indeed, in our study, we found that migratory and invasive abilities were increased in tumour spheres, compared to parental A549 cells; expression levels of N‐cadherin, vimentin, MMP‐9 and MMP‐1 were also increased in tumour spheres, compared to parental A549 cells. Cumulatively, these results indicate that tumour spheres derived from parental A549 cells possess stemness phenotypic features and may be used as lung CSCs in subsequent experiments.

Exosomes are extracellular vesicles that contain bioactive microRNAs. Recent reports have suggested that exosomes derived from the tumour microenvironment may communicate with cancer cells in the vicinity by transferring special microRNAs to regulate the metastatic potential of recipient cells. For example, CAF‐derived exosomes were found to enhance the migration and invasion of osteosarcoma cells through the transfer of miR‐1228^12^; MDSCs releasing exosomal miR‐126a were found to promote lung metastasis.^13^ In addition, mast cells may transfer exosomal miR‐490 to hepatocellular cancer cells and thus accelerate metastasis.^14^ Whether lung CSCs, as a necessary and important component of the tumour microenvironment, regulate lung cancer metastasis via the transfer of exosomal microRNAs remains to be clarified. We therefore purified lung CSC‐derived exosomes for the experiments in this study. As reported previously,^30^ the diameter of exosomes purified from lung CSCs ranged from 30 to 150 nm. This exosome population expressed special markers such as CD63, TSG101 and CD81. We labelled exosomes with PKH26 and demonstrated that these fluorescent exosomes entered lung cancer cells. We also verified that lung CSC‐derived exosomes enhanced the migratory and invasive abilities of lung cancer cells; increased N‐cadherin, vimentin, MMP‐9 and MMP‐1 expression; and decreased E‐cadherin expression. Along similar lines, Wang *et al* ^18^ reported that CSCs promoted EMT in renal cancer cells via the transfer of exosomes. 

The functional roles of miR‐210 have been described previously. As one of the major hypoxic microRNAs, miR‐210 is up‐regulated in several types of cancer including renal cell carcinoma, breast cancer, pancreatic cancer and colorectal cancer, indicating that miR‐210 may act as an oncogenic microRNA.^31^ The role of miR‐210 in lung cancer has also been reported. He *et al* ^32^ showed that miR‐210 expression was increased in cancerous tissue samples from patients with lung cancer and closely related to poor progression‐free survival in lung cancer patients. Xu *et al* ^33^ demonstrated that miR‐210 levels were up‐regulated in serum and tumour tissue from lung cancer patients and positively associated with lymph node metastasis. Indeed, some researchers have reported that miR‐210 promotes the metastasis of hepatocellular carcinoma and breast cancer cells.^34^, ^35^ Notably, there are two versions of miR‐210: miR‐210‐3p and miR‐210‐5p. Previously, only the 3’‐arm of precursor microRNA (designated as the guide strand) was thought to mature and become functional, whereas the complementary 5’‐arm (referred to as the passenger strand) was considered to be destined for degradation.^36^ For miR‐210, miR‐210‐3p is the guide strand, which integrates into the RNA‐induced silencing complex (RISC), whereas miR‐210‐5p is the passenger strand, which is inactivated by degradation. Recently, Ren *et al* ^19^ suggested that miR‐210‐3p promoted prostate cancer cell EMT and bone metastasis; Petrozza *et al* demonstrated that miR‐210‐3p may be a biomarker for clear cell renal cell carcinoma metastasis.^20^ More importantly, Xian *et al* showed that exosomes derived from hypoxic mesenchymal stem cells promoted the migration and invasion of lung cancer cells via the transfer of miR‐210‐3p.^21^ We chose miR‐210‐3p as our research target to clarify the possible involvement of miR‐210‐3p in lung cancer cell metastasis via lung CSC‐derived exosome transfer. The results of the experiments performed showed that miR‐210‐3p levels were higher in lung CSCs and in the exosomes derived therefrom, compared with control cells. Next, we transfected lung CSCs with miR‐210‐3p inhibitor or miR‐210‐3p mimic and purified exosomes derived from these exosomes to explore the functional role of exosomal miR‐210‐3p in lung cancer metastasis. As expected, our results verified that exosomal miR‐210‐3p derived from lung CSCs contributed to the pro‐metastatic phenotype of lung cancer cells, including enhanced migratory and invasive abilities as well as up‐regulated expression levels of N‐cadherin, vimentin, MMP‐9 and MMP‐1, with down‐regulated E‐cadherin expression.

Our investigation of the molecular mechanism by which lung CSC‐derived exosomal miR‐210‐3p regulates lung cancer metastasis revealed that FGFRL1 may be the functional target of miR‐210‐3p. Indeed, Liu *et al* ^37^ demonstrated that, in osteosarcoma cells, miR‐210 promoted cell migration and invasion by targeting FGFRL1. In a study of hepatocellular carcinoma, Yang *et al* ^38^ found that miR‐210 promoted cancer angiogenesis by targeting FGFRL1. Despite the tumour‐promoting roles of the miR‐210/FGFRL1 signalling axis mentioned above, some studies have yielded conflicting results. Tsuchiya *et al* ^39^ demonstrated that miR‐210 inhibited the proliferation of oesophageal squamous cell carcinoma cells by targeting FGFRL1. Zou *et al* showed that miR‐210 inhibited the proliferation of laryngocarcinoma cells and induced apoptosis through effects on FGFRL1.^40^ Recently, Yang *et al* ^41^ reported that miR‐210‐3p inhibited bladder tumour growth and metastasis by targeting FGFRL1. In this study, we demonstrated that lung CSC‐derived exosomes regulated FGFRL1 expression in lung cancer cells via the transfer of miR‐210‐3p. Furthermore, silencing FGFRL1 with siRNA promoted the migration and invasion of lung cancer cells and up‐regulated expression levels of N‐cadherin, MMP‐9 and MMP‐1, whereas E‐cadherin expression was down‐regulated. However, in FGFRL1‐overexpressing lung cancer cells, metastatic potential decreased markedly. In combination, these results suggest that FGFRL1 acts in a tissue‐ and cell‐specific manner. In lung cancer, FGFRL1 may act as a tumour suppressor, but further investigation is needed.

## 5 CONCLUSION

To sum up, our results preliminarily demonstrate that lung CSCs contribute to a pro‐metastatic phenotype in lung cancer cells by transferring exosomes carrying miR‐210‐3p. FGFRL1 may be a target of miR‐210‐3p in the regulation of lung cancer metastasis.

## CONFLICT OF INTEREST

The authors confirm that there are no conflicts of interest.

## AUTHOR CONTRIBUTIONS

Feng Luo and Li Wang conceived the study and designed the project; Li Wang performed most experiments; Jun He, Yanyang Liu and Li Tu performed some in vitro experiments; Zhen Sun and Haoyue Hu performed some in vivo experiments; and Feng Luo and Li Wang wrote and revised the manuscript. All authors contributed to the critical revision and approval of final manuscript.

## Supporting information

Fig S1

Fig S2

Fig S3

## Data Availability

Additional data and materials may be requested from the corresponding author on reasonable request.
