# Supplementary figures and images for: Lung CSC‐derived exosomal miR‐210‐3p contributes to a pro‐metastatic phenotype in lung cancer by targeting FGFRL1
Source: J Cell Mol Med. 2020 May 12;24(11):6324–39. doi: 10.1111/jcmm.15274 (PMC7294132; doi:10.1111/jcmm.15274)

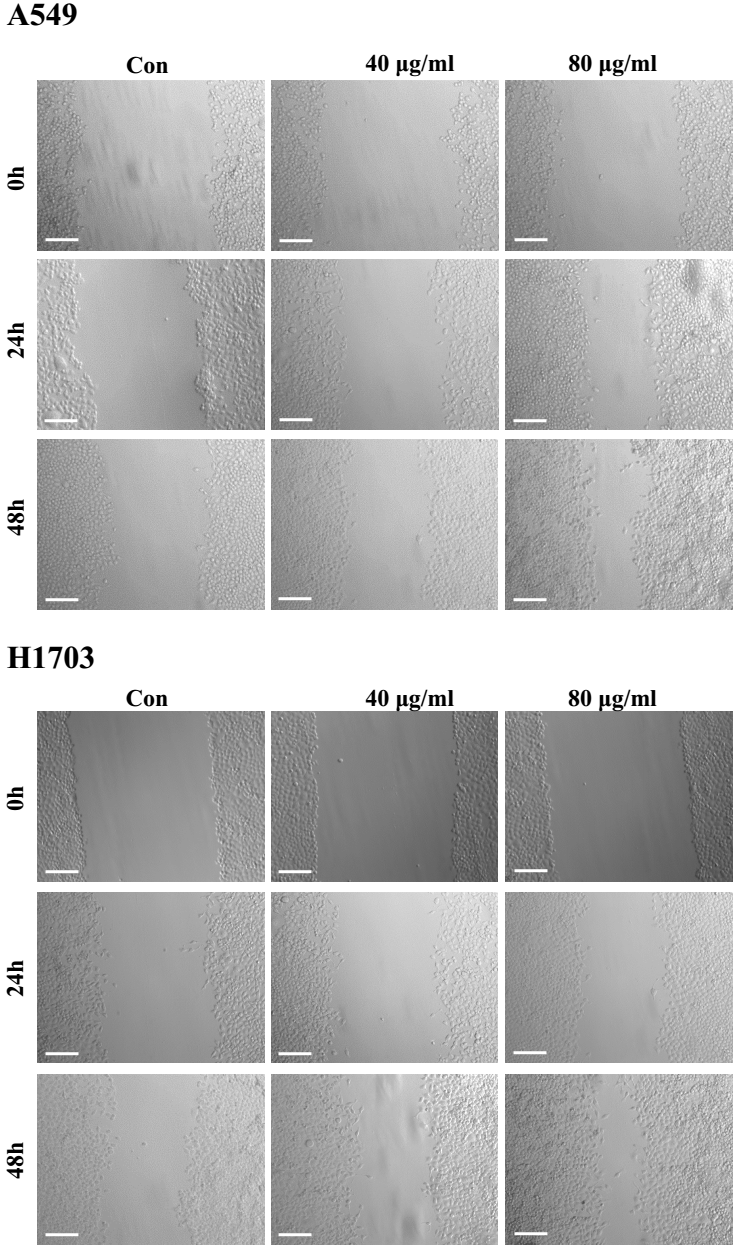

Supplement: Supplementary file 1 — Fig S1 [file JCMM-24-6324-s001.tif]

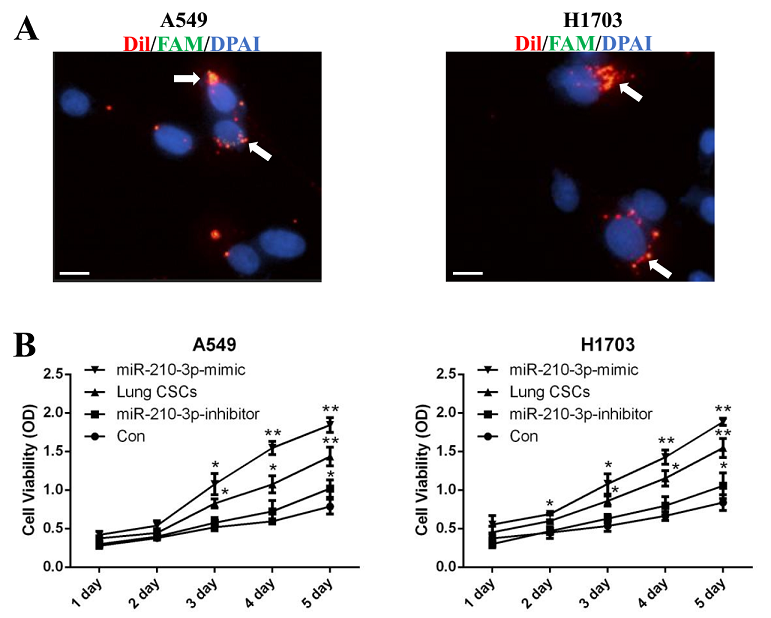

Supplement: Supplementary file 2 — Fig S2 [file JCMM-24-6324-s002.tif]

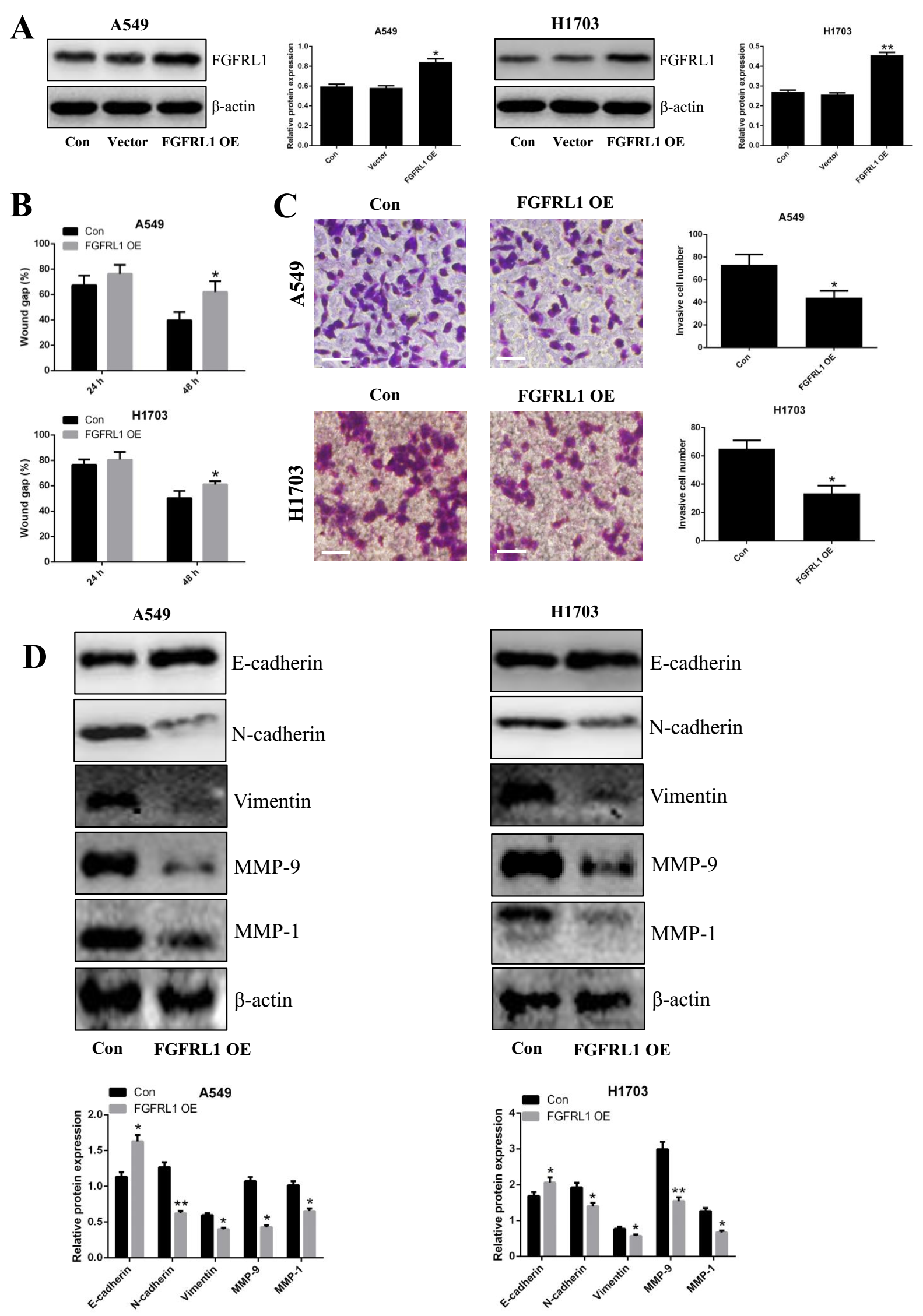

Supplement: Supplementary file 3 — Fig S3 [file JCMM-24-6324-s003.tif]
